# Decolonizing ELT teacher education by incorporating knowledge of local communities in the teaching practicum

**DOI:** 10.12688/f1000research.133704.1

**Published:** 2023-10-04

**Authors:** Luz Mary Quintero, Amparo Olarte Clavijo

**Affiliations:** 1 School of languages, Universidad Industrial de Santander, Bucaramanga, Santander, 680001, Colombia; 2 Universidad Distrital Francisco Jose de Caldas, Bogotá, Bogota, 11321, Colombia

**Keywords:** Teacher education, decoloniality, local knowledge, community-based pedagogies

## Abstract

Despite significant advances in the epistemological frameworks that guide teacher education in Colombia and elsewhere, it continues to be governed mostly by traditional Eurocentric paradigms. Decolonizing teacher education requires epistemological moves to resignify the plurality of local knowledges and praxis. This article aims at reporting a qualitative research project carried out with three student teachers of a teacher education program with emphasis on English, at a public university in the northeast of Colombia. The main objective was to explore and reflect on how EFL pre-service teachers incorporated knowledge of local communities as resources for language teaching and learning during the practicum. Data were gathered over a three-semester period through pre-service teachers’ lesson plans, materials, a final academic report, and a semi-structured interview. Data were analyzed based on the principles of thematic data analysis. Findings revealed that student teachers approached knowledge from an ecological perspective coming from different ways of knowing, seeing, being and living in the world. At the same time, the ecology of knowledges helped them to overcome the challenges they faced during the project.

## Introduction

Scholars from Colombia affirm that teacher education programs tend to be constructed around narrow, instrumental, and apolitical visions of language, language learning and teaching and are focused mainly on the traditional technical aspects of language and language teaching ( Cárdenas, González, and Álvarez-Valencia, 2010; Cárdenas, 2009; Méndez & Clavijo 2017; Sierra Piedrahita, 2016; Bonilla and Tejada, 2016). This view tends to reduce language teaching to an apolitical endeavor and fails to account for the social, cultural and historical reality of learners and teachers, their problems, motivations, aspirations, and needs. Thus, initial English teacher education programs become the ideal place for discussion and reflection on new approaches to ELT. It is at universities that pre-service teachers engage with more academic and theoretical issues as teacher education courses are in the best position if change is to come into effect ( Cavalheiro, 2016).

When reviewing the literature about higher education ( Castro-Gómez, 2007; Palermo 2014; Ortíz Ocaña, Arias López & Pedrozo Conedo, 2018) and language teacher education domains and praxis ( Borelli, Viana Silvestre, and Rocha Pessoa, 2020; Domínguez, 2019, 2020; Granados, 2016), the logic of coloniality prevails. Domínguez (2019) claims that teacher education is a process undertaken by predominantly white institutions, based on apparent discourses of diversity and multiculturalism dominated by Eurocentric thought and ideology

One of the research and pedagogical approximations to English teacher education from a decolonial perspective is “community-based pedagogies” (CPB). In Colombia, the collaborative work done by Sharkey *et al.* (2012, 2016), Clavijo and Ramirez (2019), and Salmon and Clavijo-Olarte (2022) with pre-service and in-service teachers in public schools and with teachers from private schools in the city of Bogotá is part of the social commitment and political action taken by the teacher education programs that they belong to. Clavijo considers that:

“the central aspects for language teacher education and research learned through doing community-based pedagogies are related to (1) the practice-oriented focus of the field assignments for reflection, teaching, and research; (2) the generation of local knowledge for decision-making in curriculum and teaching; (3) the transformative nature of critical and inclusive pedagogies like CBP that teaches teachers to recognize and make student and community assets the subject of curriculum (Comber, 2018); (4) and finally, the agentive role that teachers and learners take using CBP to connect school and community” (p. 51-52).

This article describes a qualitative research project using decolonial perspectives on language teacher education by implementing CBP with three practicum students at a public university in the northeast of Colombia. This project was developed during the COVID-19 pandemic (2020-2021). Our goal was to explore and reflect on *how EFL preservice teachers incorporate knowledge of local communities as resources for language teaching and learning during the practicum.* Data were gathered over a three-semester period through pre-service teachers’ lesson plans, materials, academic reports, and a semi-structured interview. The findings report that pre-service teachers approached language teaching from a decolonial perspective. They integrated different ways of seeing and being in the world by engaging with historically marginalized communities ( Domínguez, 2019).

## Theoretical considerations

When we intend to approach the concept of decoloniality, we have to necessarily refer to the concept of coloniality-modernity ( Quijano, 2000) as they constitute the two inseparable sides of the modern world system. The Eurocentric Coloniality/modernity classified human beings as superior and inferior, rational, and irrational, primitive, and civilized, traditional and modern ( Quijano, 2011). The dyad was created based on a racialized hierarchy of white Europeans and mestizos, and positioned the historical, cultural, linguistic differences and identities of the colonized as negative, inferior and even nonexistent. The imposition of such racialized Eurocentric mode of perception has had a historic impact on the production of knowledge and people’s ways of thinking and being in Latin America (Maldonado Torres, 2007; Quijano, 1992, 2007, 2011; Santos, 2006; Tlostanova & Mignolo, 2009). The global Eurocentric project related to other cultures, cosmologies, epistemologies and non-Western world views and people from a position of racial superiority ( Grosfoguel, 2011; Maldonado Torres, 2007). Castro Gómez (2005) argued that the colonizers modified cognitive, affective, and volitive configurations of the colonized people and somehow reshaped them in the image of the western beings. Therefore, the colonized naturalized the new identity and accepted the Eurocentric way of relating with natural, human, social worlds and even how they related with their own self (as cited in Ocaña, Arias López, & Pedrozo Conedo, 2017).

Quijano (2000) and Maldonado Torres (2010) differentiate between colonialism and coloniality. The former is understood as a political and economic relation in which the sovereignty of a country or nation resides in the power of a dominant country, usually a colonizer. That is the case of Latin American countries which were invaded by Spain and Portugal. The latter, coloniality, is defined as a Eurocentric mode of perception and production of knowledge, and the colonization of people’s ways of thinking and being (Maldonado Torres, 2007; Quijano, 1992, 2007, 2011; Santos, 2006; Tlostanova & Mignolo, 2009). Coloniality has survived colonialism in Latin America and is present in an imbricated system of power, being and knowledge present in all spheres of the organization of the modern world system. This imbricated system comprises economic, epistemological, subjective, gendered, cultural, and ontological dimensions ( Quijano, 2000).

The coloniality of power ( Quijano, 2007, 2011) is understood as a global hegemonic model of power that articulates race and labor, space and peoples, social class, and gender according to the needs of capital and to the benefit of white European peoples. The coloniality of being is the ontological dimension of coloniality, it points at the ‘ontological excess’ that occurs when particular beings impose on others and, beyond that the potential or actual effectivity of the discourses with which the other responds to the suppression as a result of the encounter (Maldonado-Torres 2003, 2007). Coloniality of knowledge viewed by Lander (2000) focuses on the relations of Eurocentrism and social sciences from a Latin American perspective. From a colonial view, Latin American knowledge is produced following the Eurocentric paradigms as the only valid, objective and universal ways to approach and produce knowledge. In Grossfoguel’s (2011) words “Subaltern knowledges were excluded, omitted, silenced, and/or ignored” (p. 24). The coloniality of knowledge has a central place for the purpose of the study of language and teacher education in Colombia and we will explore it in more depth in the analysis and discussion of our research findings below.

Historically, Europe and North America have been regarded as the centers of the production and dissemination of knowledge in all areas, reducing the territories of the South as not possessing a history, a coherent culture, and an epistemology worthy of recognition ( Bartlett & Vavrus, 2017). It is important to remark that these perspectives are supported by the South as most people have been educated under their hegemony ( Quijano, 2011) and therefore have accepted and internalized these forms of knowledge as the only truths. The problem with the Eurocentric frames is that they have historically subordinated, negated or made invisible ‘other’ frames, ‘other’ knowledges, and ‘other’ subjects and thinkers ( Walsh, 2005, 2007). Palermo (2021) also contends that nowadays the world continues to be organized from the logic of a global coloniality. This is evident in the complex interrelated system of beliefs accepted as the only truth that guides and organizes the world and people’s lives.

Education has not escaped the influence of global coloniality. It is one of the most powerful strategies to maintain and consolidate the global colonial power by influencing people’s minds to perceive and understand the world (Palermo 2014; Ortíz Ocaña, Arias López & Pedrozo Conedo, 2018). For the authors, pedagogical, curricular and didactic frameworks of education constitute the hidden discourse of modern coloniality. The Latin American system of education is framed within homogenizing and standardizing colonial pedagogy ( Guzmán Valenzuela, 2021). The system of education and educational processes have been traditionally oriented by local knowledge generated in Eurocentric countries and imposed as universal in the rest of the world (south) ( Ortíz Ocaña, Arias López & Pedrozo Conedo, 2018). In general, curricular contents, pedagogical strategies and teaching styles do not relate to the sociocultural and historic contexts and subjectivities of Latin America (Palermo, 2015).

The field of English language teaching is one of the areas in education that nurtures the modern system of coloniality because most of the linguistic and language learning theories, curricular plans, materials, teaching methods, standardized tests, and teacher preparation ( Kumaravadivelu, 2016) used to teach English and to educate prospective English teachers come from the “inner circle countries”. In a similar line, Granados-Beltrán (2016) claims for teacher education that includes and validates the voices of marginalized groups in education. Ramos-Holguín (2021) reflects on the need to design non-hegemonizing teacher education programs that respond to the particularities of the local realities and subjectivities, and envisions prospective teachers as transformers of the present world.

In a similar trend, Lucero & Castañeda (2021) highlight that in Colombia there is a growing well-recognized community that has been working from different fields and positions to transform ELT in the country. This community has given a central place to the locally produced knowledge in three different ways: to reconceptualize the ELT field, to resist imposed hegemonic discourses in the field, and, to value peripheral knowledge and practices. Apple (2001) agrees that English teacher education programs that intend to approach teacher education otherwise need to move away from international market-based systems that aim to create uniformity and “a system of more centralized authority over what counts as important teacher skills and knowledge” (p. 183).

Escobar (2007) argues that we need an approach to change that is “communal, indigenous, hybrid, and above all, pluriversal and intercultural” (p.11). In his words, a “gradual epistemic decolonization, understood as a long-term process of re-signification and re/construction toward words and knowledges otherwise”(12). In Santos’s (2014) view, we need a plural system of knowledges that he calls “ecology of knowledges” that value, make visible, and include the diversity of the inexhaustible world experience that cannot be accounted for by any single general theory. Palermo (2021) also claims the need to propose other ways of knowing, discovering, doing, thinking different from the ones that westernized scholarship has created and prescribed. Therefore, a larger decolonial agenda demands urgent interventions in education, curriculum, and pedagogy ( Fregroso Bailón, & De Lissovoy, 2019) so that we move towards an ecology of knowledges in education. Therefore, if we are to undertake a decolonial process in education and in the initial education of prospective English teachers, local scholars and the academic community need to give a pronounced place to the local and disciplinary discourses in the construction of local relevant knowledge ( Canagarajah, 2005).

Hsu (2017) asserts that “Initial teacher education programs possess a special potential for transforming English language teaching and for creating spaces of decolonial possibilities in the classroom” (124). So, the question is how to approach teacher education from a decolonial perspective? Pereira Borelli, Viana Silvestre, and Rocha Pessoa (2020) reflecting on some of the multifaceted challenges of decolonizing English teacher education affirm that one of the challenges teacher educators have to face is the resignification of their praxis. In other words “We [teacher educators] must dare to *live* language teacher educators *otherwise”*(p. 319). It implies that teacher educators need to move from reflection on decolonial language education towards a decolonial praxis, recognizing our praxis as political.

Reflecting on the same challenge, living teacher education otherwise, Domínguez (2019) understands that the challenge we face is not pragmatic but epistemic. He adds that “this work [living teacher education otherwise] ought to begin by grappling with the ontologies of the global south, allowing novices the epistemically difficult, but profoundly generative, opportunity to build from these new perspectives (p.54). Domínguez ideas align with the ideas of Brazilian scholars Pereira Borelli, Viana Silvestre, and Rocha Pessoa (2020) in the sense that we need to move to praxis. He is concerned about how teacher educators help preservice teachers to articulate decolonial discourse and reflection with their future day-to-day work at school, “especially when the challenges we hope they will embrace—disrupting oppression and advancing liberation—are so complicated” ( Domínguez, 2020, p.8). In his view, teacher education programs need to offer pre-service teachers concrete models of decolonial repertoires of practice in order to make sure that teacher education prepares future practitioners to advance the decolonial project in education. This implies that decoloniality is not only reflected in the teachers’ evolving conceptual understanding, but, essentially, in the emergence of decolonial classroom practices ( Kubanyiova, 2012).

From our understanding of a decolonial perspective on education, we propose CBP as one possible option to approach English teacher education from a decolonial perspective. Different pedagogical experiences and research projects have been carried out by teacher educators to explore and document how language teachers understand, develop and implement community-based pedagogies during the last 15 years ( Clavijo & Ramírez, 2019; Clavijo-Olarte & González, 2016; Flórez González, 2018; Nieto, 2018; Sharkey, *et al.*, 2016; Rincón & Clavijo Olarte, 2016; Gómez, 2018; Sharkey & Clavijo-Olarte, 2012; Clavijo-Olarte & Austin, 2023). Research and pedagogical results have pointed out that this approach increases teacher awareness of deficit discourses in education and promotes respect and value for local communities, their knowledge, practices, and cultures; strengthens relationships and feelings of co-existence among the different community members; incorporates communities as a vital part of the curriculum; engages pre-service teachers, school students and community members in critical action and reflection to transform their situated realities; promotes professional, learning autonomy and creativity; creates opportunities for the co-construction of knowledge; and fosters the natural use of different linguistic and semiotic repertoires available for communication.

Similar initiatives using CBP with pre-service teachers have been carried out at other public universities in Colombia ( Bolaños, *et al.*, 2018; Lastra, *et al.*, 2018; Hernández Varona & Gutiérrez Álvarez, 2020). Among other findings, their studies show new avenues to conceive the practicum as a reflective practice that reconceptualizes pedagogy and curriculum. A critical reflective practice that involves pre-service teachers in observation and analysis of the local resources while learning the foreign language is not centered exclusively in Eurocentric materials but on locally situated materials produced by teachers to learn English beyond vocabulary, grammar and the development of language skills. We believe that English teaching requires understanding the personal, social and political dimensions of language(s) to engage learners in a diversity of learning activities through language.

## Methods

This interpretative qualitative study explores the realities and experiences of three student teachers in their practicum. For Merriam (2002), “Qualitative researchers are interested in understanding and learning how individuals experience and interact with the social world and the meaning it has for them” (p.4). We use an ecology of knowledges ( Santos, 2014) perspective to study socio-political, cultural, and social dimensions of the topics uncovered by student teachers in their teaching of historic memory and identity with language students. We intend to explain sociocultural and political issues of teaching localized in the study of two topics: historic memory and identity that were of interest to student-teachers. Our explanation is constructed from our understanding and analysis of curricular sequences, the materials designed to guide their students to become aware of the value of local historical resources, the interview we carried out with them, and the final written report.

### Context and participants

This study was carried out at a public university in the northeast of Colombia offering a tenth-semester Bachelor in foreign languages with emphasis on English. The participants of this study were three student-teachers (two women and one man), who were purposefully selected because they were doing their teaching practicum with an emphasis on community-based pedagogies. Participants’ ages ranged between 21 and 24 years old. Participants who were not doing their teaching practicum within the concept of community-based pedagogies were not included. One of the authors was the student teachers’ mentor or supervisor of the practicum and the second author was an observer. Both researchers actively participated in the data collection and analysis process. According to the Education Act 02041, Practicum has become a central part of teacher education programs in the country. Pre-service teachers start their practicum earlier in their programs (before they have finished the first 50 credits of the program). Following the policy, the Pedagogical practicum at the university where the study took place is divided in three stages: Observational practicum (3 to 5 semesters), guided practicum (6 and 7 semesters), and innovation practicum (8, 9 and 10 semesters).

The research project took place in the last stages of the practicum (innovation): “Pedagogical Practicum” I, II and III in the last three semesters of the program, during the second semester of 2020 and the academic semesters of 2021. In Practicum I, students carried out an analysis of the educational context, based on the outcomes of the analysis, student-teachers designed two pedagogical projects that were implemented, analyzed, and evaluated in Practicum courses II and III. Due to the emergency caused by COVID-19 pandemic and the difficulty to be in school contexts, the Practicum was done with the students enrolled in the English courses (pre-intermediate, intermediate and upper intermediate) in the same teacher education program. All the process took place through the platform Zoom, whose access was granted by the university.

Regarding community-based research experiences at the University, it is relevant to mention that teacher researchers belonging to the language teacher education programs at the school of languages at the university have conducted research that illustrates the impact of utilizing the human, linguistic, social, ecological and cultural resources in local communities. Their research and pedagogical goals were to explore, understand and analyze social and cultural issues that become part of the content of English courses and foster interdisciplinary work among professors from different programs at the university. Several professors from other programs (Social work, Laws, History, Civil engineering) were invited as expert guest speakers to illustrate students and teacher educators in the topics that they intended to explore in the English courses. Guest speakers give their specialized talks either in English or in Spanish.

### Instruments for data collection

*Semi-structured interviews*

We designed and piloted a questionnaire for the semi-structured interview and administered it to the three participants. The authors designed the entire questionnaire based on the purpose of the project and the central tenets of community-based pedagogies and decolonial theories. The individual interviews took around 40 minutes each and we invited participants to join us through Google Meet. Each interview was audio-recorded and transcribed afterwards. We designed a questionnaire that contained five questions that would provide us with details about the ways they incorporated knowledge of local communities as resources for language teaching and learning during the practicum. Semi-structured interviews are considered by Horton *et al.* (2004) to be flexible both in designing and refining the interview questionnaire and in conducting the interviews. They believe that semi-structured interviews allow “the interviewees a degree of freedom to explain their thoughts and to highlight areas of particular interest and expertise that they felt they had”. The authors contend that “this form of interviewing also revealed certain issues that they had not previously identified, and which could be followed up in further questioning/as well as in later interviews” (p.340).

*Curricular units*

Curricular units consisted of the long-term planning that preservice teachers designed to sequence the main topics (identity and historical memory) that were addressed during the two academic semesters that lasted the implementation of the two pedagogical projects. Two curricular units were included in the study. The curricular unit’s chart designed by the researchers was used as a guide for students to structure their practice. Student teachers selected the themes considering the results of the needs analysis carried out in Practicum I. It addressed seven aspects that guided the pre-service teachers in their planning: stages & time, themes, objectives, learning resources, learning outcomes, language focus, and assessment. These seven aspects aimed at explaining to the pre-service teachers how to integrate local resources with language and literacy activities, language functions, grammar and lexis to engage students and help them achieve the learning outcomes. The curricular unit was the main source for pre-service teachers to design the lesson plans and materials used during the development of the pedagogical projects.

*Final report*

This document contains the description, implementation, analysis, and evaluation of the pedagogical projects undertaken during the teaching practicum. This document allowed researchers to know the rationale that underlies pre-service teachers’ decisions; theoretical, pedagogical, and didactic frameworks; as well as the main findings and conclusions they drew from the pedagogical projects. The final report was a group assignment presented by student teachers at the end of Practicum III.

*Reflective journals*

Student teachers’ reflective journals were used to document the way they experienced practice and dealt with situations in their practicum. Hubbs & Brand (2010) define reflective journaling as a method for enhancing understanding of course content, a strategy for making meaning, and a means for illuminating and critiquing student understanding.

### Ethical considerations

Retrospective ethical approval was obtained from the Council of the School of Languages at Universidad Industrial de Santander, as recorded in the minute No. 16 session number 16, on May 26, 2023. At the beginning of the research process, researchers obtained written informed consent from the three participants, given the fact that the study was classified as “no risk” according to Resolution 8430 of the Colombian Ministry of Health, since this project does not include any sensible information about the conduct, personal information of the participants and will not harm participants physical or psychological integrity. In order to comply with the publication policies of the journal, we retrospectively sought and obtained ethical approval. In order to obtain informed consent, researchers invited the three student teachers to participate in the study. First, we contacted each participant by phone individually and explained the purpose of the study and the type of data that would be collected, as well as the instruments for data collection. We also explained that their participation was voluntary and did not represent any risk to their physical or mental safety. We also explained the procedures we would use to guarantee their anonymity, as well as their autonomy and freedom to withdraw from the study at any moment. The three of them accepted to be part of the project and signed the written consent.

**Data analysis**

We gathered the data from the three participants through online sessions during the year 2021. As we mentioned above our instruments were semi-structured interviews, the curricular units designed by the participants and their final reports. We organized the data into files and created folders in google drive to have the data available for analysis. We used a thematic analysis approach to identify recurring instances of the responses to the interview questions as well as the content of the curricular units and final reports. Thematic analysis is described by Braun and Clarke (2006) as “a flexible and useful research tool, which can potentially provide a rich and detailed, yet complex account of data” (p. 5). The researchers read and coded the data to discuss the emergent themes and to construct the final emerging categories that are discussed below.

The themes that emerged from the analysis revealed connections between student teachers’ didactic knowledge coming from English-speaking countries and local knowledge produced by Colombian scholars; the design and selection of learning materials that aimed at including voices of actors who experienced violence, discrimination, or exclusion; and the use of both languages English and Spanish as sources for learning and teaching. Considering the plurality and complementarity of factors that the themes revealed and informed by the theory of ecology of knowledges by Santos (2014), we titled the first category approaching knowledge from an ecological perspective; and the second category problematizing ELT to situate practice and make visible the local for teaching and learning.

**Findings**

In this section, we intend to discuss the findings through two categories followed by a brief definition of each one. Then, the reader will find the analysis and discussion of each category supported by samples of the data that illustrate the main points that we want to highlight.

### Approaching knowledge from an ecological perspective

Colonial/modern models of monocultural thinking and knowledge prevail in today’s thinking and practice including university settings. The decolonial movement has claimed for the recognition of other forms of knowledge present in the global south. For Santos (2014) global social justice is intimately linked to global cognitive justice. Therefore, the struggle for global social justice must be a struggle for global cognitive justice too. Global cognitive justice makes visible suppressed, marginalized, or silenced knowledge and allows the copresence and coexistence of different types of knowledge. In other words, Santos (2014) advocates for an ecology of knowledges. In the data analysis process, researchers identified that preservice teachers recognized and included different types of knowledge in all the stages of the practicum. Below, we will discuss and illustrate these points.

The analysis of student teachers’ reflections and experiences during the practicum provided us with a wealth of situations that show them involved in multiple processes that implied decision-making for designing lessons, curricular sequences, teaching materials, and activities to engage students during the implementation. As mentioned by student teachers in the individual interviews, they devoted a great deal of time to finding relevant sources that included printed, visual, and multimodal materials and invited expert guest speakers (as suggested by the practicum professor) to inform the two projects. Student teachers included knowledge from different academic sources, disciplines, and community members to promote critical reflection on the main topics. We evidenced how student teachers approached knowledge from an ecological perspective first by including didactic knowledge coming English speaking countries and local knowledge produced by Colombian scholars. Second, by observing student teachers design and select learning materials that include voices of actors who have experienced violence, discrimination, or exclusion. Third, the ecology of knowledge was manifested using both languages English and Spanish (translanguaging) as sources for learning and teaching.

First, student teachers recognized that mainstream didactic knowledge about English teaching (mainly coming from England and the USA) and knowledge produced by local scholars and teachers became complementary sources of knowledge for the design, creation, implementation, and evaluation of their practicum projects. In the following quotation from an interview, participant 2 stressed the importance of local knowledge in the design of the curricular units:

“We read a lot about CBP, we read a literature review and we read studies and I think for us it was very important the experience that other educators had had regarding this topic. That was very important for us besides the mere concepts. We also read about experiences that other teachers had had and we looked for resources and projects that other teacher educators had carried out” (Participant 2, 3:53-4:27).

The curricular unit, the lesson plans and the materials also depicted knowledge of mainstream didactics as essential for the design and the implementation stages of the projects. Student teachers approached speaking and writing with ease as competent users of the language and exhibited methodological knowledge that permitted them to scaffold the production of multimodal written and oral texts. Student teachers’ decisions focused on providing students with a sequence of activities through examples and relevant input that prepared them to create the final texts: Digital TED talks and digital storybooks. Thus, teaching about what a TED talk was by watching TED talks and analyzing the structure; presenting and explaining the key elements of a ted talk; improvising storytelling; teaching how to narrate past events; and guiding the creation of the TED talk were part of the process of guiding learners in the producing of their TED talks. This can be exemplified in the following excerpt.

“For the first one we used several videos about a TED talk because the final outcome was a Ted talk that students needed to do, and yes ted talks we were looking at short stories or in some classes, we helped them learn how to do an interview, write a story, to learn how a fictional story is created, the parts of this ….” (Participant 1, interview, 4:51’-5:20’).

**Figure 1.**
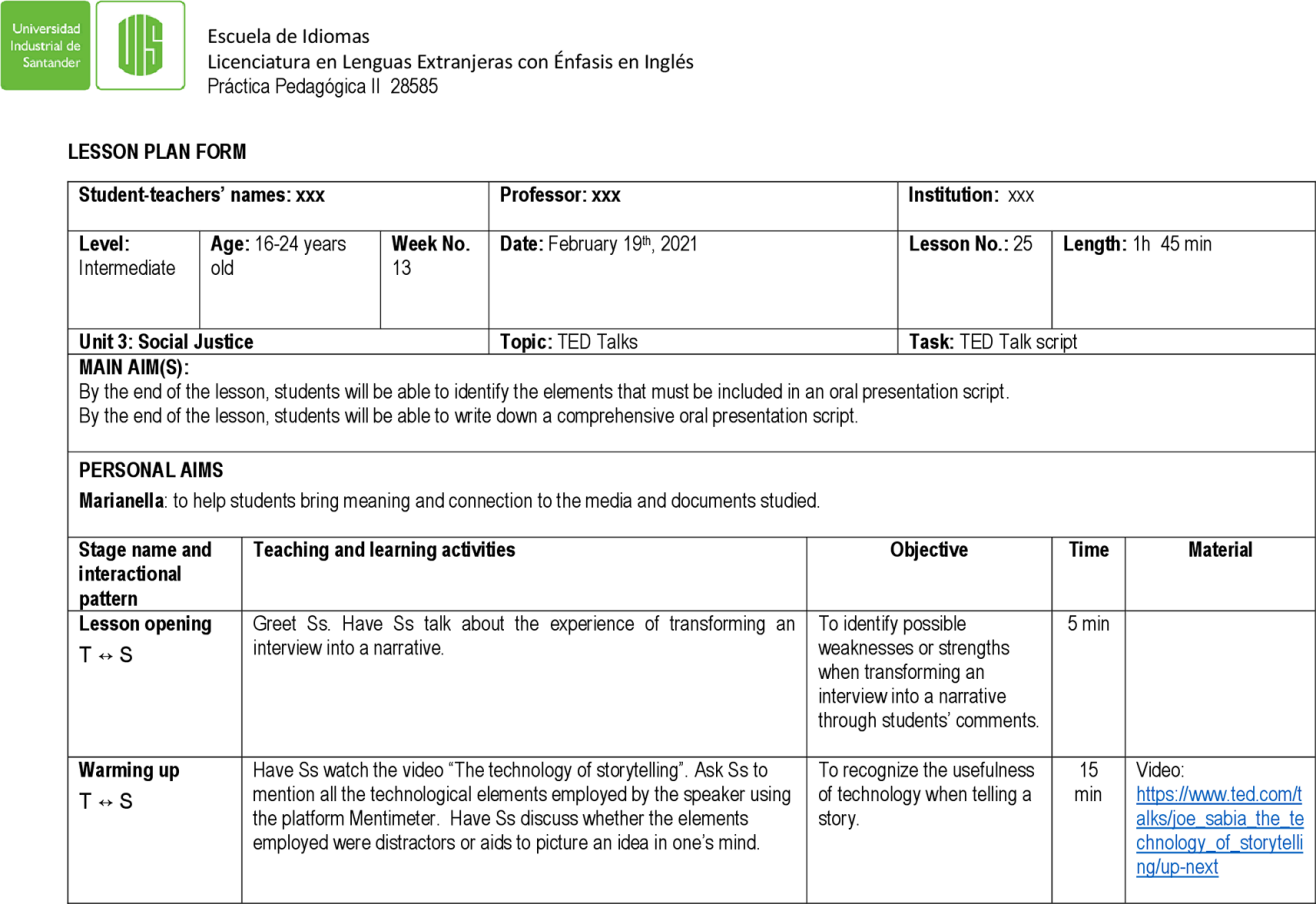
Chart lesson plan 21, February 5, 2021.

Figure 1, an extract of lesson 21 is an example of the chart that student teachers used to plan the lesson objectives, teaching activities, and materials, to carry out a class. The lesson aimed at discussing social justice as the topic of the unit, while enhancing oral skills and familiarizing students with key elements of TED talks.

Grammar also played an important role in the projects as student teachers needed to make decisions about the levels of complexity of vocabulary (simple to sophisticated ways of expressing ideas) and grammatical patterns. They designed diverse and situated learning experiences and materials that helped learners to make meaning in more complex and elaborated ways and supported them in the creation of the multimodal texts. Evidently, grammar was at the service of creating TED talks and digital storybooks to tell stories of the lived experiences of exclusion, discrimination, and violence in Colombian communities.

Second, an ecological perspective of knowledge was present in the learning materials designed or selected by student teachers in order to include the voices of several actors who have approached violence, discrimination or exclusion from different lived experiences and viewpoints. This diversity offered a rich variety of knowledge coming from photographers, historians, artists, justice leaders, victims of violence and local community members. The knowledge of different actors was incorporated in the projects through expert guest speakers, friends and relatives, historical readings (Archivo Nacional de Memoria Histórica), documentaries, stories told by the victims of violence and photography. The previous voices became the main sources of knowledge for the learners to generate discussion and reflection on the key topics from multiple local perspectives and at the same time to learn the language.

“…when implementing, which was in practicum II and practicum III, we used the information of historical memory here in Colombia, the armed conflict, the peace process … we made students do interviews and do research about this…with the input we gave them, the videos, the conversation, and books that we used … we saw some stories, we saw also Abad Colorado’s photographies, and some videos of the armed conflict, a little bit about peace process and then they needed to create a story based on the arm conflict here in Colombia. They needed to invent a story but for example with a real person, a real place of war, for example Bojaya … or something like that.”

(Participant 1: Interview: 1:52-3:59)

In the final report, student teachers reflected on how knowledge of community members represented in the stories of the lived experiences were vital to help their learners to broaden their perspectives of exclusion, discrimination, and violence:

***“*** Considering learners were encouraged to reflect on their identities, a pertinent strategy to engage them with the community was to work under the premise that everyone has a story to tell. Through these activities, students were able to appreciate different social situations that affect different communities and social groups like stereotypes, prejudices, discrimination, violence, social problems, and how such influence their identities and life stories … The intention of such endeavors was to raise awareness about unequal social dynamics while listening to different testimonies as well as to highlight how these social aspects take place in society and in our closest contexts” (Group final report, page 20).

“…We discussed different types of identities, social, cultural and identities that are inherited in the family and cultures, these identities. In the ted talks, I could see that, for example, one student talked about a lesbian girl. One student talked about a trans girl that was a friend of hers. So, they portrayed in these stories the identities of the person sexually” (Participant 3, Interview, p.5).

Finally, student teachers designed and used a variety of multimodal materials for meaning-making to complement the traditional written and oral ways to communicate in the language classroom. Through TED Talks, photography, documentaries, and films student teachers provided information to reflect on discrimination, social groups, identity, and Colombian historical memory. They were also used as examples for students to create their own TED talks to denounce the experiences of discrimination lived by people in their communities. This can be seen in the digital stories that learners produced (Story 6).

They (the students) needed to create a story whose focus was related to the conflict. So, we read some stories, we learned also about color, and some photography and some videos of their conflict and the peace process are a little bit about the peace process. They needed to create a story based on the conflict here in Colombia, they needed to invent a story. But with, for example, a real person or a real place, or a real event that happened in Colombia. For example Bojayá, or something like that (Participant 3: interview p.1).

**Figure 2.**
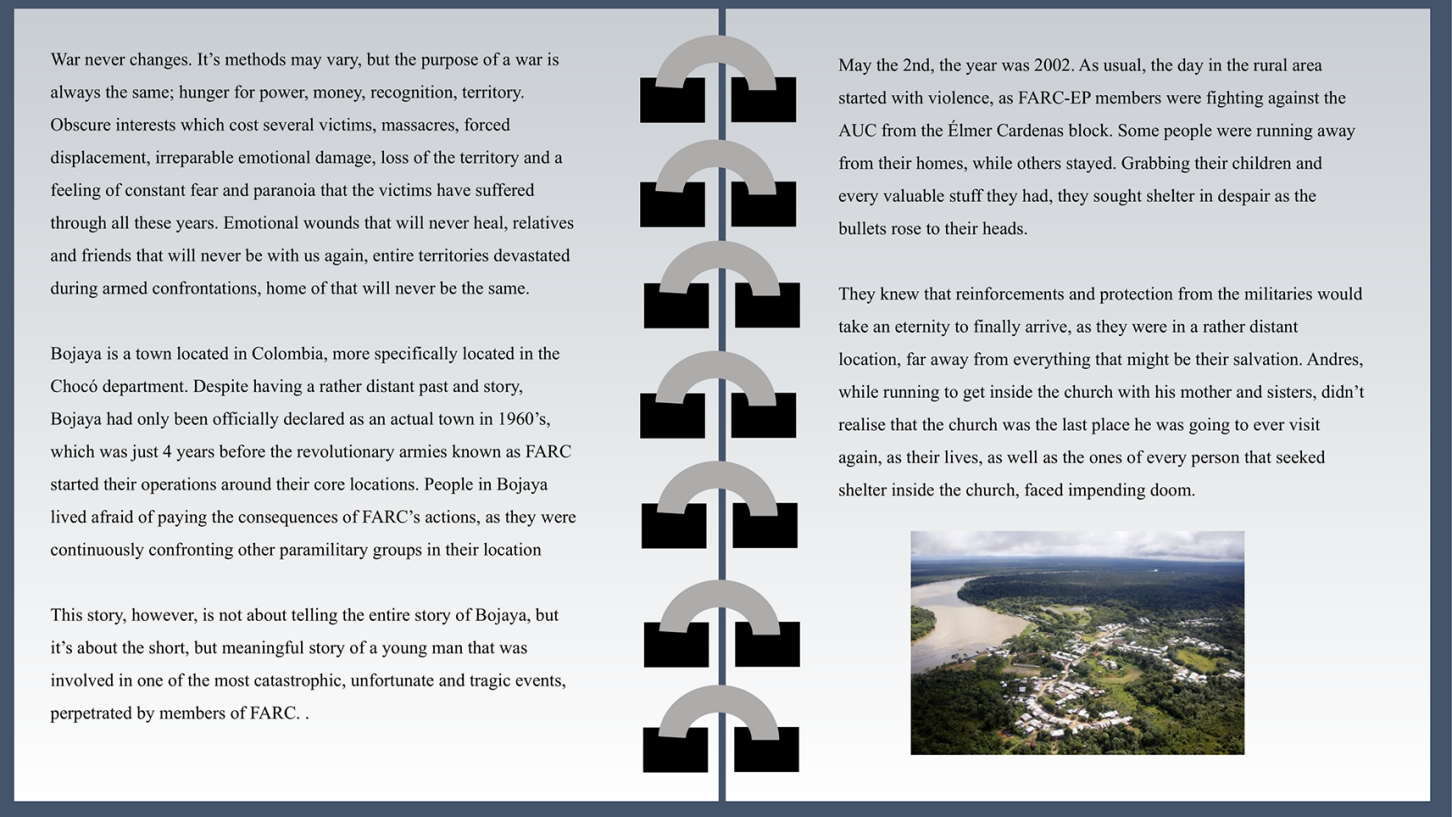
Story 5. Note: The text and images are published with prior written consent from the author of the story who also consented to publish this figure. The names of the characters in the story are fictional but are associated to a real massacre that took place in Colombia in 2002, in Bojayá, Chocó.

The story ( Figure 2) depicts the situation of a Wayuu indigenous girl in the north of Colombia.

Thirdly, another way to exemplify the ecology of knowledge is using both languages English and Spanish as sources for learning and teaching. Student teachers’ decision to use material and human resources that used Spanish as their first language and English as the target language evokes Ruiz’s (1984) theory about language as a societal resource. It works as an invitation to teachers to view languages as material resources in classrooms that contribute to expanding and understanding local knowledge as relevant to decolonize the school curriculum. Local expert guest speakers were an important human resource for expanding students’ learning of the topics studied. In this case the talks and the interactions took place in Spanish, so the language was not seen as a problem but as a resource to enrich the knowledge of the topics and complement the language-learning process.

More recently, scholars consider translanguaging as a complex process of interweaving languages in second or foreign language contexts. Li (2017) cited García (2009) to make her claim visible that “translanguaging empowers both the learner and the teacher, transforms the power relations, and focuses the process of teaching and learning on making meaning, enhancing experience, and developing identity” (15). We believe that in teaching and research, it is possible to use material, linguistic and human resources that come in Spanish or English to document a given topic. Materials for teaching were available in Spanish and in English and student teachers used them as they were implementing their classes. It is illustrated below.

“We found this book, this book is available in Spanish and in English, so we used the English version … the photographies …. and the description of the pictures in this book was in Spanish and they needed to read it and then to translate it in order to take parts for their stories …”

(Participant 1, interview 6:50-7:15)

Likewise human resources as guest speakers and experts were key in the development of the pedagogical activities as they enhanced the understanding of the topics developed from a local perspective. These experts from different specialized fields, available at the university and social organizations were invited by student teachers to share their knowledge and perspective of the local situations.

“There was another guest, there were some guests, and they talked about historical memory, strikes and different things. They spoke in Spanish because I guess they can´t have a conversation totally in English and the students also responded to this talk in Spanish and then we turned into English.”

(Participant 1, interview: 7:45-8:14)

As illustrated in the category above, the information for the design of the curricular units and materials as well as for the implementation of the projects came from different yet complementary sources of knowledge. The recognition of and dialogue among different knowledges represented an ecology of knowledges. Thus, knowledge of mainstream didactics for foreign language teaching, mainly Eurocentric, was enriched with the one coming from the situated praxis of teachers and researchers in the Colombian context. Knowledge presented in official books, publications, documentaries, and the academic discourses was complemented with knowledges coming from the voices of ordinary people (victims, friends, relatives) who have lived situations of exclusion and violence. In the same direction, both native and foreign languages were viewed as rich resources in the different stages of the teaching practicum.

This plurality of knowledges represented different ways of knowing, being, seeing and interpreting the world from loci of enunciation. It also permitted student teachers to construct and understand teaching as weaving different experiences to help language learners to critically adopt an informed position and at the same time advance in the learning of language. For Pereira Borelli, Viviane Silvestre and Rocha Pessoa’s (2020, p.304), facing the challenges of teacher education otherwise, we need to “strive for collectiveness, plurality, and locality”.

### Problematizing ELT to situate practice in making visible the local for teaching and learning

The teaching practicum at the university where the project took place is conceived as a stage in which student-teachers are expected to propose innovative projects for language learning. In the context of this research project, the teacher educator started by raising awareness about the importance of taking a closer look at the local (whose knowledge is valuable?; What problems do local people have? What cultural sources are visible?, how do people use language and what languages are visible in the communities?). By problematizing the concept of the local and its role in ELT, student teachers could identify and analyze to what extent the local resources have been invisible or narrowly understood and limited to a shallow view of learners’ likes and interests suggested in textbooks (hobbies, popular music, fashion, famous people, movies) and expand their view to include the context in more disruptive ways.

Escobar (2007) defines the local as concrete places in which people make sense of the world and from which knowledge emerges. For the author, local knowledge is the individuals’ mode of place-based consciousness. In the ELT field the local has also gained visibility, especially in the critical perspectives of the last decades. Kumaravadivelu (2003) from a postmethod perspective, remarks the centrality of the local, considering that it can generate location-specific, classroom-oriented innovative strategies for practitioners. In the light of the three principles of the postmethod pedagogy, particularity, practicality and possibility ( Kumaravadivelu, 2003, 2006), Clavijo and Ramírez (2019) emphasize the relevance of associating learning with the local dynamics and lived experiences of the learners, relating it to the pedagogical knowledge that emerges from the daily practices of teachers, and creating environments that involve the students’ knowledge to explore with them their identity and ideologies, in order to position it in a social and political context.

Designing lessons to teach EFL using the local knowledge to explore the topics from a critical perspective was the approach we used to guide curriculum design and provide feedback about teaching to the student teachers in the regular feedback sessions with the mentor. As we mentioned above, problematizing ELT to situate it in the local realities implied focusing the practicum on aspects beyond the teaching of language skills. It required placing practice in the realities of students´ communities and inquiring about questions like whose knowledge is valuable; What problems do local people have? What cultural sources are visible?, how do people use language and what languages are visible in the communities? As students expressed it below, connecting language teaching with social and political realities of people in Colombia that implied gathering data from people in the communities about the armed conflict, historical memory, identity and exclusion provided genuine reasons to learn in classrooms.

“Yes, it was really challenging because this planning (Practicum II) is really different from what we were used to, and we had to start from nothing … because when we are not just teaching the topics they are learning (grammar) like we are used to because when we teach, we get a curriculum” (Interview: Participant 3, p. 6)

Regarding the topics, we have touched sexual orientation and in these last days we have touched the one of gender identity among others. I love these two topics specifically and the way the students have managed them and responded to them make me feel very glad and proud of them since they are really critical people in a good sense … bias, stereotypes, prejudices, and discrimination are other topics we have seen in the last days, and they are getting new knowledge in regard to these concepts and understand them very well. (Reflective journal, Participant 3, p. 1).

During the planning stage of the second action plan it was questioned the fact whether students would have “close” experiences regarding the Colombian armed conflict. There was no doubt that all of the events that occurred in Colombia have shaped our society in one way or another; nonetheless, it was difficult to know before implementing the project whether students or their family members would have experienced such events. To our surprise, few learners did have memories about that period of time, for most it was not their stories, but their relatives’ and acquaintances’. (Reflective journal, Participant 1, p. 2).

Problematizing ELT also implied questioning the poor level of engagement, fostering inquiry skills, Information and Communication Technologies (ICT) use, individual and group participation and language production learners are expected to carry out in the language classroom. We envisioned from the beginning of the practicum involving students in extensive reading of multimodal texts and different genres, writing of multiple texts such as critical reflections, multimodal digital books, creative writing, TED talks, videos, and reports. Their texts revealed the expanded view of teaching a language and placing local knowledge at the core of the teaching practice and the commitment of teacher educators and student teachers in constructing a new experience in their practicum.

**Figure 3.**
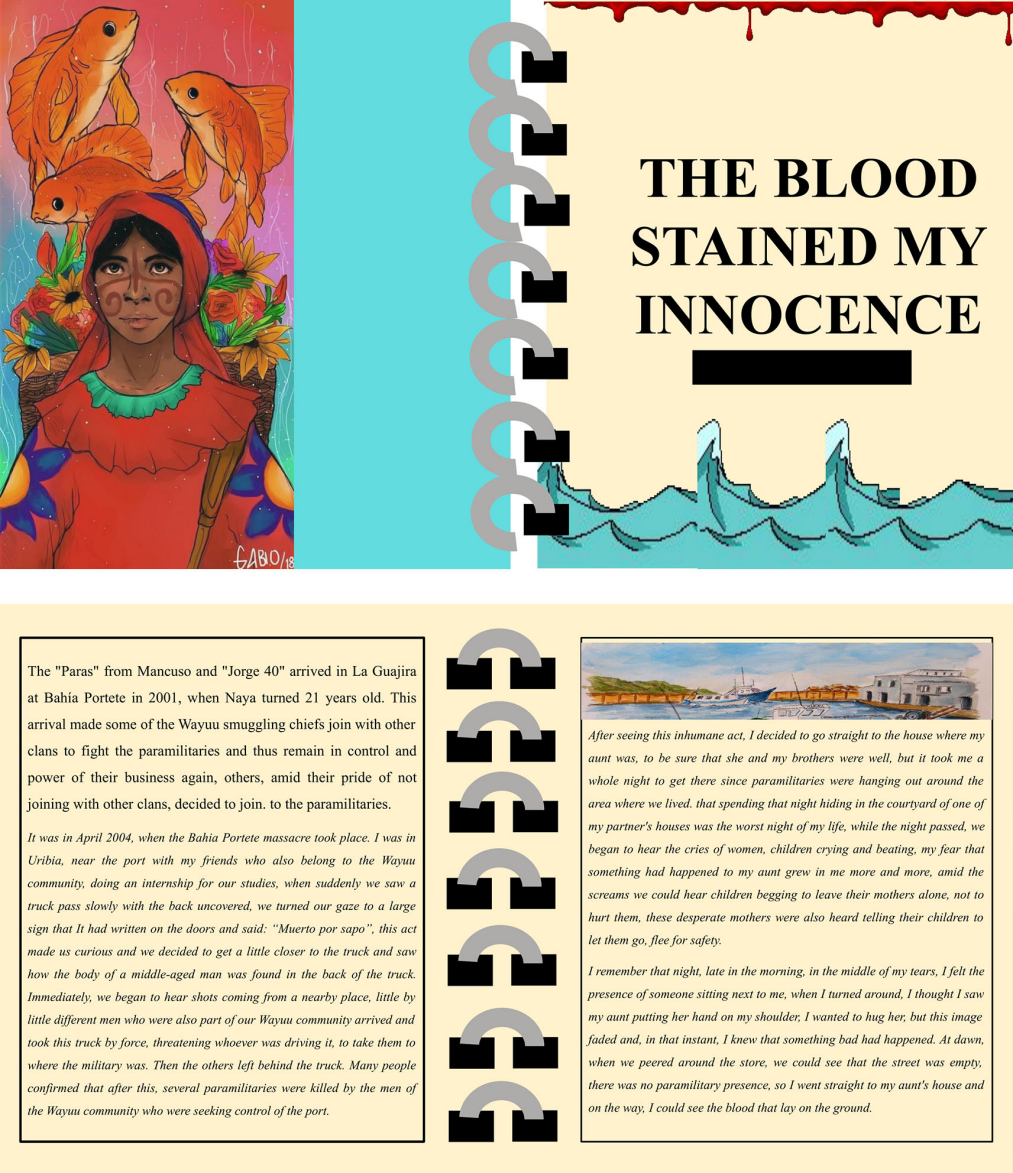
Story 4. Note: The text and images are published with prior written consent from the two authors of the story, who also consented to publish this figure. The names of the characters in the story are fictional but are associated with a real massacre that took place in 2004 in Bahía de Portete, Guajira-Colombia.

In their writing of digital stories, students’ texts revealed the ways they became acquainted with many instances of violence in Colombia. Student-teachers presented different materials gathered from people’s narratives, documentaries, photographs, and local experts. Students created stories about the armed conflict describing the violent events using fictional and real characters in their digital stories as shown in the story ( Figure 3).

Learning to teach using multiple resources available in the educational context brought about successful experiences and challenges for student teachers in the implementation of the two pedagogical projects. They felt they were successful in teaching EFL by engaging students in experiences through topics that were critical to them.

## Conclusions and final reflections

This interpretive qualitative research examined the practicum experience of three student teachers to report the ways they incorporated knowledge of local communities as resources for language teaching and learning. Through the information collected during a year from student teachers’ action plans, the final reports, individual interviews, and reflective journals, we analyzed the way they designed, implemented, and reflected on the value of the local in ELT. Student teachers viewed the classroom as a window to project possibilities to view the ways that society positions identities revealed by different social groups that were discriminated against. In studying the topics of identity and Colombian historical memory through different sources of knowledge, student teachers unveiled real situations lived by victims that revealed social injustice and discrimination. Such issues motivated critical discussions around documentaries, publications and through interaction with experts that informed their digital stories in which their students represented the role of the victims in the Colombian war.

Learning how to incorporate local resources in teaching languages was a process that was initiated by reading and discussing professional readings by national scholars whose works illustrate ways to understand their positioning about the local. The readings provoked discussions and challenged student teachers to think of ways to integrate knowledge of topics and linguistic knowledge to help their students advance as critical readers and writers.

Thus, they carefully selected materials available and created new materials to address together the topics chosen by the projects. By using the languages available in the local documents, the classroom, the city, the multimodal materials, the university experts and other local experts as resources for learning, students constructed their understanding of the event studied in the language class. Finally, student teachers promoted critical literacies through reading, discussions and writing of narratives that depicted stories of war in Colombia and the construction of identities that are invisible in Colombian society.

Finally, as stated at the beginning of the article, English teacher education is a process undertaken by white or whitened institutions and dominated by Eurocentric epistemology ( Domínguez, 2019). Therefore, teacher education programs need to build spaces where subjectivities, voices and the plurality of local subaltern knowledges are unveiled, recognized and made visible. A decolonial project such as that requires collaboration, a set of concerted and coordinated actions that value local action and knowledge, and cherish community, plurality and collaboration ( Borelli, *et al.*, 2020; Castañeda Trujillo, 2018; López-Gopar, 2014). It means including other epistemic locations from which reality is thought instead of rejecting or negating Eurocentric knowledge, through a blind acceptance of it ( Mignolo and Walsh, 2018). In other words, it requires epistemological and ontological moves (Silvestre, 2016) so that we, teacher educators can resignify our praxis and dare to live English teacher education otherwise ( Pereira Borelli, Viana Silvestre and Rocha Pessoa, 2020).

Finally, the English teacher practicum built on emerging decolonial frameworks need to offer student teachers practice-based experiences that help them to translate the discursive decolonial repertoires into rich decolonial repertoires of practice ( Domínguez, 2019). Obviously, we do not refer to proposing decolonial models or methodologies, we refer to pragmatic and relational skill sets that will help future English teachers to operationalize the decolonial and teach English otherwise. We believe that community-based pedagogies can offer possibilities to decolonize teacher education praxis.

## Data Availability

Figshare: INTERVIEW TRANSCRIPTS, https://doi.org/10.6084/m9.figshare.24030210.v1 ( Quintero, 2023a). This project contains the following underlying data:
- Interview 1. docx. - Interview 2. docx. - Interview 3. docx. Figshare: REFLECTIVE JOURNALS.pdf, https://doi.org/10.6084/m9.figshare.24030201.v1 ( Quintero, 2023b). This project contains the following underlying data:
- Journal 1.pdf. - Journal 2.pdf. - Journal 3.pdf. Figshare: ACTION PLAN, https://doi.org/10.6084/m9.figshare.24046986.v1 ( Quintero, 2023c). This project contains the following underlying data:
- Action Plan Intermediate.pdf (Lesson plan) Figshare: FINAL REPORT, https://doi.org/10.6084/m9.figshare.24047022.v1 ( Quintero, 2023d). This project contains the following underlying data:
- Final Report. Prácticum II pdf. Figshare: INTERVIEW PROTOCOL, https://doi.org/10.6084/m9.figshare.24046959.v1 ( Quintero, 2023e). This project contains the following extended data:
- Interview protocol.pdf Data are available under the terms of the Creative Commons Attribution 4.0 International license (CC-BY 4.0).
