# Supplementary material for: Decolonizing ELT teacher education by incorporating knowledge of local communities in the teaching practicum
Source: F1000Res. 2023 Oct 4;12:1264. [Version 1] doi: 10.12688/f1000research.133704.1 (PMC10632588; doi:10.12688/f1000research.133704.1)
Supplement: Peer review history

# Reviewer report: Reviewer response for version 1

**DOI:** 10.5256/f1000research.146714.r216219

**Published:** 2023-11-08

**Authors:** Amanda Thomas

**Affiliations:** 1 University of Nebraska–Lincoln, Lincoln, Nebraska, USA

**Summary**

 As succinctly stated in the manuscript, “This article describes a qualitative research project using decolonial perspectives on language teacher education by implementing CBP with three practicum students at a public university in the northeast of Colombia.” While this reviewer cannot claim a deeply nuanced command of the literature base for this study, the authors do an excellent job constructing and conveying the research and theory upon which their study is based. While overall strong, more detailed description of how data were analyzed would improve the paper. The study is timely and important and the authors draw conclusions that inform the nexus of research and practice.

**Points to address**

- Write out all terms before using acronyms. EFL, ELT, etc.
- Why were identity and historical memory the topics selected for this study? Adding a brief description of the topics and rationale for their choice would strengthen the paper. This might be included in the “Context and Participants” or “Curricular units” sections of the paper.
- In Paragraph 2, Sentence 3, under the Context and participants section, please clarify “the students”. Does this refer to the student-teachers, fellow students in the teacher education program, or K12 students who they were teaching via Zoom in their practicum?
- Elaborate on how the thematic analysis was carried out. The authors could improve the description of data analysis by addressing questions such as: Were all data sources analyzed independently by each researcher? Was analysis split between the two researchers and, if so, how was interrater reliability established? Was there a single level of coding or multiple iterations of coding to arrive at the emergent themes?
- It reads as though there were three themes: 1. Connections between student teachers’ didactic knowledge coming from English-speaking countries and local knowledge produced by Colombian scholars; 2. the design and selection of learning materials that aimed at including voices of actors who experienced violence, discrimination, or exclusion; and 3. the use of both languages English and Spanish as sources for learning and teaching. Yet, two categories are titled. I am surmising that the two categories draw from the three themes and the theory of ecology of knowledges, but please provide further clarify for readers. This might also be addressed through more detailed elaboration of the data analysis, as previously mentioned.
- In the fourth sentence of the second paragraph in the “Approaching knowledge from an ecological perspective” section, the word “from” should be added between “coming English”. Also, while the intention is obvious, the next sentence is technically not a complete sentence.
- Be consistent with capitalizing “TED talk”.
- Provide more support or evidence for the claim, “Evidently, grammar was at the service of creating TED talks and digital storybooks to tell stories of the lived experiences of exclusion, discrimination, and violence in Colombian communities.”

Is the work clearly and accurately presented and does it cite the current literature?

Yes

If applicable, is the statistical analysis and its interpretation appropriate?

Not applicable

Are all the source data underlying the results available to ensure full reproducibility?

Yes

Is the study design appropriate and is the work technically sound?

Yes

Are the conclusions drawn adequately supported by the results?

Yes

Are sufficient details of methods and analysis provided to allow replication by others?

Partly

Reviewer Expertise:

Teacher education, STEM education, mathematics education, technology in mathematics teaching and learning

I confirm that I have read this submission and believe that I have an appropriate level of expertise to confirm that it is of an acceptable scientific standard.

# Reviewer report: Reviewer response for version 1

**DOI:** 10.5256/f1000research.146714.r213259

**Published:** 2023-11-08

**Authors:** Rosane Rocha Pessoa

**Affiliations:** 1 Universidade Federal de Goias, Goiânia, State of Goiás, Brazil

**Brief summary**

 The article titled “Decolonizing ELT teacher education by incorporating knowledge of local communities in the teaching practicum”, by Luz Mary Quintero and Amparo Olarte Clavijo, reports an interpretive qualitative research project carried out with three student teachers of a teacher education program with emphasis on English at a public university in the northeast of Colombia. Its main objective was to reflect on how EFL student teachers incorporated knowledge of local communities as resources for language teaching and learning during their practicum. The research material was generated over a three-semester period by means of a semi-structured interview with the student teachers, their lesson plans, their final academic report, and their reflective journals. The student teachers addressed two topics in their classrooms, identity and Colombian historical memory through different sources of knowledge, which resulted in their students’ production of digital stories that depicted stories of war in Colombia and the construction of identities that are invisible in Colombian society. The findings were discussed in two themes, “Approaching knowledge from an ecological perspective” and “Problematizing ELT to situate practice in making visible the local for teaching and learning”, showing that working with local knowledges, praxis, and translanguaging is an important move towards decolonizing language teacher education.

**Overall opinion**

 The article is a relevant decolonial undertaking in the area of English teacher education because it breaks with traditional Eurocentric paradigms in many aspects. First, because it brings principles from “community-based pedagogies” (CPB), which had already been researched by Colombian scholars, including the second author of this article, Amparo Olarte Clavijo, and which are coherently linked to Decolonial Thought in the article. Second, because student teachers decided to work with Colombian’s social, cultural and historical reality in their classrooms. Third, because it focused on praxis (I would replace the two terms by the term “local praxiologies” since I think they can only be seen entangled), local knowledges, and translanguaging. The theoretical background is comprehensive and sound, in which important concepts of Decolonial Thinking are discussed (modernity/coloniality; colonialism and coloniality; coloniality of power; coloniality of being; coloniality of knowledge) and related to education, English language teaching, English language teacher education and initial teacher education. What is highlighted in this section is the need to focus on teachers’ praxis in order to pluralize knowledges in our area so that less oppressive and more just ways of knowing, doing, thinking can be created. In the findings, the authors present a very good discussion on how the student teachers included different types of knowledge in all the stages of the practicum by intertwining mainstream pedagogical knowledge and local knowledge produced by Colombian scholars, by highlighting the student teachers’ materials that included voices of actors who have experienced violence, discrimination, or exclusion, and by using both English and Spanish (translanguaging) as sources for learning and teaching. Decolonially speaking, I believe it is of great relevance that learning was associated “with the local dynamics and lived experiences of the learners, relating it to the pedagogical knowledge that emerges from the daily practices of teachers, and creating environments that involve the students’ knowledge to explore with them their identity and ideologies, in order to position it in a social and political context” (p. 11).

**Detailed discussion and suggestions**

 I have some concerns and suggestions and they are divided into major and minor comments. The major comments are related to the Methods and the minor comments concern all sections of the article.

**Major comments**

 In the Context and participants, there are only two sentences related to the participants: “The participants of this study were three student-teachers (two women and one man), who were purposefully selected because they were doing their teaching practicum with an emphasis on community-based pedagogies. Participants’ ages ranged between 21 and 24 years old”. However, I think that if we are to confront colonialities in our research, besides focusing on teachers’ praxis, we should also address their subjectivities/bodies. I do not think it is enough to mention their gender and their age. It is important to describe/problematize their identities in terms of race, class, profession (Were they only students? Did they work?), political views on Colombia, conceptions about English and English language teaching, etc. Besides, I think they should have names (they could be fictitious names chosen by them) instead of being referred to as Participants 1, 2, and 3. I believe that if we aim at confronting colonialities in our research we should start working with the concept of embodied knowledge, in which the body is understood as “living and historical territories which allude to a cosmological and political understanding where our wounds, memories, knowledges, desires, and individual and common dreams reside” (Cruz Hernández, 2017, p. 43).

 The research instruments are mentioned differently in the article. In the Abstract and in the Introduction, it reads: “lesson plans, materials, a final academic report, and a semi-structured interview” (three instruments). In the Methods, the instruments for data collection are described as: “semi-structured interviews, curricular units, final report, and reflective journals” (four instruments). In the Data analysis, it is affirmed that the authors use a thematic analysis approach to identify recurring instances of the responses to the “interview questions as well as the content of the curricular units and final reports” (three instruments). In the Conclusions and final reflections, they are described as: “action plans, the final reports, individual interviews, and reflective journals” (four instruments). I suggest that they are worded the same and appear in the same order in the four times they are referred to in the article. I also suggest that they are mentioned in order of importance.  

**Minor comments**

 In the Abstract, it reads, “the research material was generated ‘over a three-semester period’”, while in the Conclusions and final reflections it reads “Through the information collected ‘during a year’ from student teachers’ action plans, the final reports, individual interviews, and reflective journals […]”.

 The term “teacher education” is used many times in the text. In the first sentence in the abstract it is stated: “Despite significant advances in the epistemological frameworks that guide ‘teacher education’ in Colombia and elsewhere, it continues to be governed mostly by traditional Eurocentric paradigms”. I understand the authors may want to refer to it generally, but in some cases I wonder if it should not be “English teacher education” instead.

 In the Semi-structured interviews, it reads that the questionnaire contained five questions. I suggest that the questions are stated in this section. I am aware that the data (or the research material, term of my preference) is available in the Data availability, but I had a look at the interviews and the questions are not easily grasped, so I think it would be elucidative if the questions were stated objectively in this section.

 In the Curricular units, it reads: “The curricular unit’s chart designed by the researchers was used as a guide for students to structure their practice. Student teachers selected the themes considering the results of the needs analysis carried out in Practicum I”. Here, the authors affirm that the curricular unit’s chart was designed by the researchers, but in the Data Analysis, 2 ^nd^ line, they affirm that the curricular units were designed by the participants. I also have two questions concerning this extract are: Could this curricular unit’s chart be made available to the readers? Which themes were selected by the student teachers?

 In the Semi-structured interviews and in the Reflective journals, these instruments are defined generally according to some authors, but these definitions are not related to what has been done in the research: 1) “The authors contend that “this form of interviewing also revealed certain issues that they had not previously identified, and which could be followed up in further questioning/as well as in later interviews” (p.340)”. Concerning this quote, my question is: was there further questioning or later interviews in the study? 2) “Hubbs & Brand (2010) define reflective journaling as a method for enhancing understanding of course content, a strategy for making meaning, and a means for illuminating and critiquing student understanding”.  Regarding this quote, my question is: Did the reflective journals serve this purpose in the study?

 In the Final report, it reads: “The final report was a group assignment presented by student teachers at the end of Practicum III”. Was this final report done by the group of the three research participants?

 In Approaching knowledge from an ecological perspective, it is affirmed that students created two types of final texts: Digital TED talks and digital storybooks. However, only digital storybooks are shown in the article. I wonder if there should be an explanation concerning that. Besides, different terms are used to describe them (“digital books”, “digital storybooks”, and “digital stories”).

 Also, in Approaching knowledge from an ecological perspective, the Ficture 2 caption is

 “The story (Figure 2) depicts the situation of a Wayuu indigenous girl in the north of Colombia”, but her story is in Figure 3.

 In regard to the stories in Figure 2 and 3, I think it is important to mention if the texts are shown the way they were written by the students or if they have been edited.

 Although the text is well-written, it needs careful textual and formatting revision. I will give just one example concerning formatting. The following reference appears differently each time it is mentioned: Borelli, Viana Silvestre, and Rocha Pessoa, 2020; Pereira Borelli, Viana Silvestre, and Rocha Pessoa (2020); Pereira Borelli, Viviane Silvestre and Rocha Pessoa’s (2020, p.304), Borelli, et al., 2020; Pereira Borelli, Viana Silvestre and Rocha Pessoa, 2020. Besides, most references in the article include just the last name.

**Article-specific questions**

 I have answered “yes” to the questions “Are sufficient details of methods and analysis provided to allow replication by others?” and “Are all the source data underlying the results available to ensure full reproducibility?”. However, I do not think they are applicable to interpretive qualitative research such as the one discussed in the article. The research was an attempt to decolonize ELT teacher education by focusing on knowledge produced both locally and collectively by researchers, student teachers and their students in the classrooms, so I do not see how it can be replicated under the same – or similar – circumstances so that prior results can be confirmed. As a matter of fact, each classroom is unique and would imply different lesson plans, different projects, different themes, different activities and different responses from the students.  

**References**

 CRUZ HERNÁNDEZ, DT. **Una mirada muy otra a los territorios-cuerpos femeninos.** *Solar*. 2017; 12(1): p. 35–46.

Is the work clearly and accurately presented and does it cite the current literature?

Yes

If applicable, is the statistical analysis and its interpretation appropriate?

Not applicable

Are all the source data underlying the results available to ensure full reproducibility?

Yes

Is the study design appropriate and is the work technically sound?

Yes

Are the conclusions drawn adequately supported by the results?

Yes

Are sufficient details of methods and analysis provided to allow replication by others?

Yes

Reviewer Expertise:

Applied Linguistics and English Language Education

I confirm that I have read this submission and believe that I have an appropriate level of expertise to confirm that it is of an acceptable scientific standard, however I have significant reservations, as outlined above.

## References

[1] : Una mirada muy otra a los territorios-cuerpos femeninos. *Solar* .2017;12(1) :35-46

# Reviewer report: Reviewer response for version 1

**DOI:** 10.5256/f1000research.146714.r213258

**Published:** 2023-11-07

**Authors:** Rosa Alejandra Medina

**Affiliations:** 1 University of Massachusetts Amherst, Amherst, Massachusetts, USA

This study responds to the dearth of literature regarding decolonial approaches in ELT from a global South perspective. The curriculum units presented are compelling and clearly illustrate this epistemological approach in practice.

 In the methods section it was mentioned that the study follows and interpretative qualitative analysis according to Merriam. Somehow the description of the approach appears succinct, and the procedures follow for data collection are not explicitly described.  I suggest:

1. Complementing the rationale for the approach
2. Adding a paragraph and even a figure or table where the stages for data collection are clearly stated. Also including the number of participants, curriculum units analysed, and timeline.
3. Clarifying how the epistemological approach “ecology of knowledges” may be related to an interpretative qualitative design. This relationship is not that obvious for the reader in the methods section. Perhaps ecology of knowledges belongs to the introductory or theoretical foundations orientation.

In terms of data analysis method, it would be appropriate to justify why thematic analysis was used, even when data are presented multimodally.

 In the conclusion section as an addition, it would be interesting to see a short summary of the pedagogical implications (So what), bullet points  for TESOL education programs,  tin preparation and in service  teachers.

Is the work clearly and accurately presented and does it cite the current literature?

Yes

If applicable, is the statistical analysis and its interpretation appropriate?

Not applicable

Are all the source data underlying the results available to ensure full reproducibility?

Yes

Is the study design appropriate and is the work technically sound?

Yes

Are the conclusions drawn adequately supported by the results?

Yes

Are sufficient details of methods and analysis provided to allow replication by others?

Partly

Reviewer Expertise:

Decolonizing approaches, qualitative post – qualitative research, applied linguistics, TESOL, translanguaging, multimodality.

I confirm that I have read this submission and believe that I have an appropriate level of expertise to confirm that it is of an acceptable scientific standard, however I have significant reservations, as outlined above.
